# Female Sexual Preferences Toward Conspecific and Hybrid Male Mating Calls in Two Species of Polygynous Deer, *Cervus elaphu*s and *C. nippon*

**DOI:** 10.1007/s11692-015-9357-0

**Published:** 2015-11-23

**Authors:** Megan T. Wyman, Yann Locatelli, Benjamin D. Charlton, David Reby

**Affiliations:** Mammal Vocal Communication and Cognition Research, School of Psychology, University of Sussex, Falmer, BN1 9QH UK; Muséum National d’Histoire Naturelle, Réserve de la Haute Touche, 36290 Obterre, France; School of Biology and Environmental Science, University College Dublin, Belfield, Dublin 4, Ireland; Department of Wildlife, Fish and Conservation Biology, University of California, 1331 Academic Surge Building, One Shields Ave., Davis, CA 95616 USA

**Keywords:** Sexual communication, Vocalization, Species discrimination, Mating, Sexual preference, Hybridization, Introgression, Deer

## Abstract

The behavioral processes at the basis of hybridization and introgression are understudied in terrestrial mammals. We use a unique model to test the role of sexual signals as a reproductive barrier to introgression by investigating behavioral responses to male sexual calls in estrous females of two naturally allopatric but reproductively compatible deer species, red deer and sika deer. Previous studies demonstrated asymmetries in acoustic species discrimination between these species: most but not all female red deer prefer conspecific over sika deer male calls while female sika deer exhibit no preference differences. Here, we extend this examination of acoustic species discrimination to the role of male sexual calls in introgression between parent species and hybrids. Using two-speaker playback experiments, we compared the preference responses of estrous female red and sika deer to male sexual calls from conspecifics versus red × sika hybrids. These playbacks simulate early secondary contact between previously allopatric species after hybridization has occurred. Based on previous conspecific versus heterospecific playbacks, we predicted that most female red deer would prefer conspecific calls while female sika deer would show no difference in their preference behaviors toward conspecific and hybrid calls. However, results show that previous asymmetries did not persist as neither species exhibited more preferences for conspecific over hybrid calls. Thus, vocal behavior is not likely to deter introgression between these species during the early stages of sympatry. On a wider scale, weak discrimination against hybrid sexual signals could substantially contribute to this important evolutionary process in mammals and other taxa.

**Electronic supplementary material:**

The online version of this article (doi:10.1007/s11692-015-9357-0) contains supplementary material, which is available to authorized users.

## Introduction

Hybridization and subsequent introgression are important evolutionary processes that can directly impact biodiversity (Seehausen 2004; Mallet 2007). Since 10–30 % of plants or animals hybridize and exchange genes (Abbott et al. 2013), it is critical to examine how these processes arise within the context of species discrimination and mate choice. Species-specific mating signals play an important role in both intraspecific mate assessment and interspecific species discrimination (West-Eberhard 1983; Bradbury and Vehrencamp 2011; Panhuis et al. 2001; Coyne and Orr 2004). Individuals often evaluate intraspecific mating signals in order to select mates which may ultimately increase their reproductive success (Andersson 1994). Additionally, species receptiveness to mating signals can act as a pre-zygotic reproductive isolating mechanism (Dobzhansky 1937; Mayr 1963) that prevents interspecific matings which are often, but not always, costly to individual fitness (Rhymer and Simberloff 1996; Barton 2001; Burke and Arnold 2001).

Most species react more strongly to signals from conspecifics than non-conspecifics (Andersson 1994; Catchpole and Slater 1995; Ryan et al. 2007). However, there is great variation in the strength and direction of this response pattern (reviewed in Ord and Stamps 2009) and sex- or species-based asymmetries in interspecific mating responses are not uncommon (Gerhardt 1974; Ryan and Wagner 1987; Cobb et al. 1988; Patton and Smith 1993; de Kort et al. 2002; Pfennig and Simovich 2002; Svensson et al. 2007). Reactions to non-conspecific mating signals can be impacted by a range of phylogenetic, phenotypic, ecological, demographic, and individual factors (see Wirtz 1999; Ord et al. 2011; Willis 2013 for review). For example, high similarity between signals may impede the ability to differentiate between conspecifics and non-conspecifics (Grant and Grant 1997; de Kort et al. 2002; Willis et al. 2014). Furthermore, phenotypically intermediate hybrid signals (as seen in insects, Mousseau and Howard 1998, anurans, Gerhardt 1974, fish, van der Sluijs et al. 2008, birds, Derégnaucourt et al. 2001; de Kort et al. 2002, mammals, Long et al. 1998; Page et al. 2001) may facilitate introgression by further reducing successful species discrimination. Regardless of a reaction’s basis, when species do not solidly discriminate against reproductively compatible heterospecifics or hybrids, hybridization or introgression are more likely to occur (Randler 2002).

Inaccurate species discrimination is likely to be a key driving factor for introgression in many instances of early secondary contact between species. Since behavior plays such a strong role in hybridization and introgression (Grant and Grant 1997; Willis 2013), examining behavioral responses to heterospecific and intermediate hybrid mating signals during the early stages of contact between reproductively compatible species can provide important insights into the emergence of these important evolutionary processes. Previous behavioral studies on this topic involving mate choice have focused strongly on fish (e.g., Rosenfield and Kodric-Brown 2003; Verzijden et al. 2012), anurans (e.g., Littlejohn and Watson 1976, Höbel and Gerhardt 2003), arthropods (e.g., Blows and Allan 1998), and birds (e.g., Derégnaucourt and Guyomarc’h 2003), with less focus on mammalian species (e.g., see Shurtliff 2011 for review). In species with genetically determined mating signals that are relatively stereotypical, anatomically constrained, and sexually selected, such as the sexual vocalizations of many mammals, we would expect these signals to present a robust barrier to hybridization and introgression. Especially in females where the cost of reproduction is commonly higher than in males. However, recent studies on reproductively compatible red deer, *Cervus elaphus,* and sika deer, *C. nippon,* indicate that sexually selected male calls may not fully deter hybridization stemming from female choice during early stages of secondary contact between previously allopatric populations despite wide differences in call properties (Wyman et al. 2011, 2014). Here, we further our investigation into behavior-driven sexual isolation in mammals by assessing the behavioral response of estrous female red deer and sika deer to male mating calls from conspecifics and hybrids.

Red deer and sika deer are closely related species (Ludt et al. 2004; Pitra et al. 2004) that exhibit large phenotypic differences in both appearance (e.g., coloration, antler configuration, and size, with red deer approximately twice the size of sika deer) and select behaviors (e.g., mating vocalizations) (Geist 1998). These sexually-dimorphic species are naturally allopatric but the introduction of sika deer into Europe since the nineteenth century has resulted in localized hybridization and introgression with free-ranging native red deer (Harrington 1973; Putman and Hunt 1993; Abernethy 1994; Goodman et al. 1999; Diaz et al. 2006; Bartoš 2009; McDevitt et al. 2009; Senn and Pemberton 2009; Biedrzycka et al. 2012; Smith et al. 2014). Successful interspecific crosses can be reciprocal (Ratcliffe 1987; Senn and Pemberton 2009) and although initial hybridization is rare, introgression is more extensive as fertile hybrids can backcross with either parent species (Goodman et al. 1999; Senn and Pemberton 2009; Senn et al. 2010a).

One of the largest phenotypic differences between these species is the loud sexual call produced by males during the reproductive season as they establish and defend harems of females or territories where females assemble (Clutton-Brock and Albon 1979; Miura 1984; Carranza et al. 1996). The acoustic properties of these mating calls are thought to be sexually-selected through the mechanisms of male competition and mate choice (McComb 1987, 1991; Minami and Kawamichi 1992; Reby et al. 2005; Charlton et al. 2007; Reby et al. 2010). Despite functional similarities, the temporal and spectral properties of these calls are considerably different between the parent species and are intermediate in red × sika hybrids (Long et al. 1998). Overall, red deer ‘roars’ are relatively short, low in fundamental frequency (F0), and are delivered in multi-call bouts (mean call duration = 1.9 s, Kidjo et al. 2008; mean F0 = 106.9 Hz, F0 range 61.7–136.8 Hz, Reby and McComb 2003) while sika deer ‘moans’ are longer, higher in frequency, and are produced as single calls (mean duration = 4.36 s; F0 range 196–1187 Hz, Minami and Kawamichi 1992). Previous experiments examining the behavioral reactions of estrous female Scottish red deer (*C. e. scoticus*) and Japanese sika deer (*C. n. nippon*) in response to paired playbacks of male mating calls from both species showed asymmetries in species discrimination abilities: most red deer hinds preferred calls from conspecific males over calls from heterospecific sika deer males (Wyman et al. 2011), while sika deer hinds showed high variability in responses with no significant difference in preferences directed towards either species (Wyman et al. 2014).

Here, we compare the behavioral responses of estrous Scottish red deer and Japanese sika deer hinds to paired presentations of mating calls from unfamiliar conspecific males versus novel red × sika hybrid males (“unfamiliar” = no prior experience with these particular conspecific males, “novel” = no prior experience with any hybrid individual). Based on the previous results from conspecific versus heterospecific playbacks, we predict similar asymmetries will persist between these species. Specifically, we predict red deer hinds will express more preference behaviors towards conspecific calls over hybrid calls, while sika deer hinds will show no difference in preference behaviors directed at these two call types. Attention behaviors were also examined in these experiments.

## Materials and Methods

### Study Overview

**Fig. 1:**
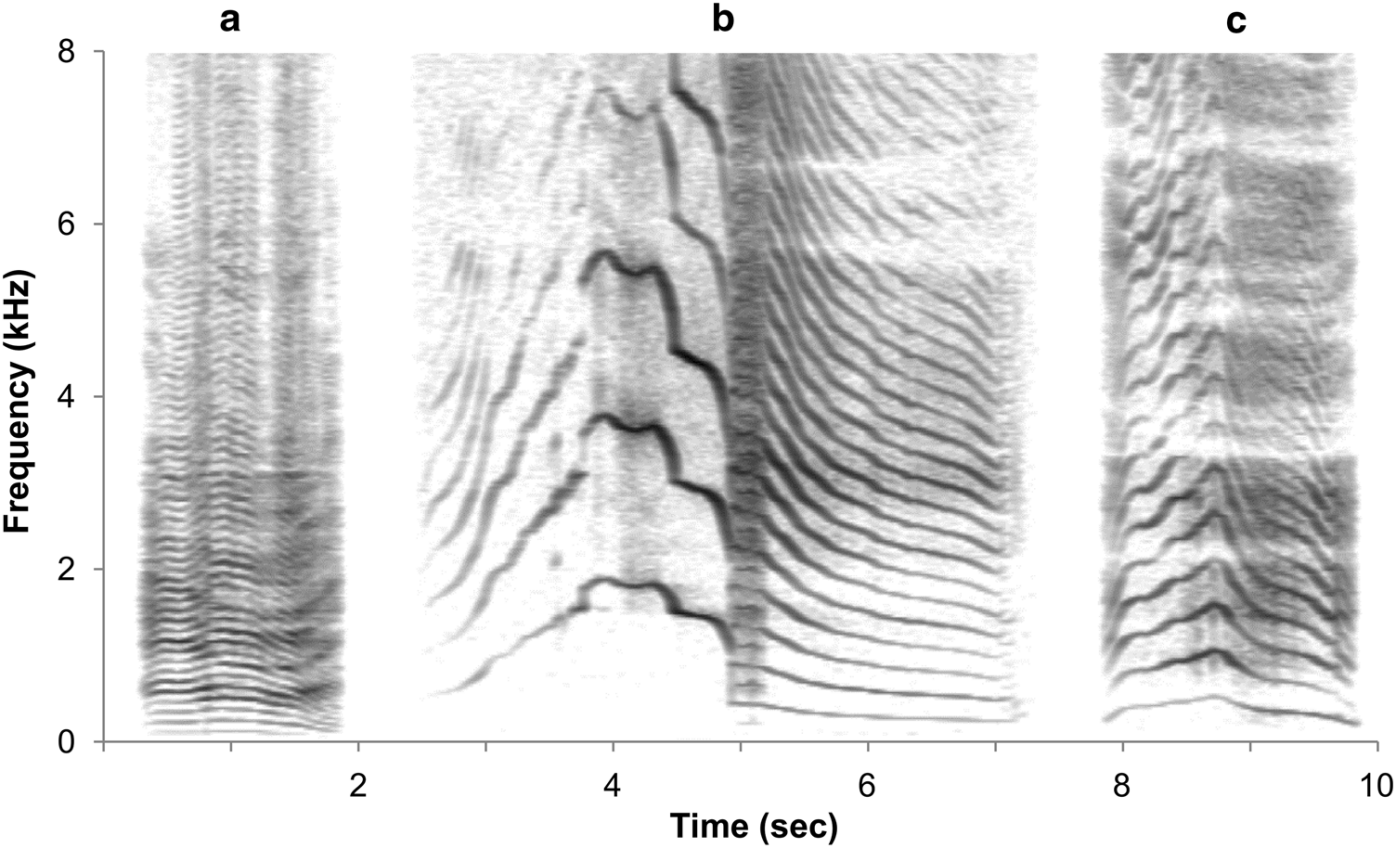
Spectrogram of adult male mating vocalizations: **a** red deer roar, **b** sika deer moan, and **c** red × sika F1 hybrid deer wail

Playback experiments on captive populations of Scottish red deer and Japanese sika deer hinds were conducted at La Haute Touche, Obterre, France. The functionally equivalent male mating calls used as acoustic stimuli in these experiments included red deer ‘roars’, sika deer ‘moans’, and red × sika hybrid deer ‘wails’ (Fig. 1; Online Resource 1). Eighteen red deer hinds (ages 2–8 years old) were presented with conspecific male red deer roars versus male hybrid wails on September 21–22, 2011. Sixteen sika deer hinds (ages 2–5 years old) were exposed to conspecific male sika deer moans versus male hybrid wails on November 23–24, 2011. All individual exemplar males were unfamiliar to the focal female subjects. Furthermore, none of the focal females had any previous contact with the other species or hybrids. As such, hybrid male vocalizations were defined as novel to these subjects. The estrous cycles of focal females were synchronized so that playbacks could be conducted during peak estrus. All females used in these experiments had previous mating experience with conspecifics. Additionally, there is no evidence of previous hybridization within the experimental populations. All experiments were conducted in accordance with the Association for the Study of Animal Behavior/Animal Behavior Society guidelines for the ethical use of animals in research, and were carried out in accordance with the procedural and ethical authorization of the French Government (DDCSPP authorization number C-36-145-002 to Parc La Haute Touche).

### Estrus Synchronization

Synchronization was achieved by inserting intra-vaginal sponges (sika deer: 1 × 45 mg, red deer: 2 × 45 mg, Intervet, Angers, France) impregnated with flurogestone acetate (FGA). The sponges provided a steady, continuous release of progesterone that inhibited normal hormone cycling by preventing follicular growth and the subsequent release of estradiol. Eight days after sponge insertion, 75 (sika deer) or 150 µg (red deer) of cloprostenol (Estrumate) were administered with intramuscular injections. The sponges were removed 12 days after insertion and females were subsequently injected intramuscularly with 100 (sika deer) or 400 UI (red deer) of eCG (equine Chorionic Gonadotropin) in order to induce estrus and ovulation. Playback trials were conducted 35–48 h after sponge removal and PMSG injection, during the predicted window of peak estrus (see Reby et al. 2010 for details). As all trials could not be performed in one day, females in each experiment were randomly split into equal groups, with Group A undergoing the estrus synchronization procedure and subsequent trials one day before Group B.

### Playback Stimuli

The acoustic stimuli used in playback experiments consisted of sexual calls from 12 individual adult males from three types of exemplars: four Scottish red deer, four Japanese sika deer, and four red × sika hybrid deer. Male red deer roars were recorded at the Institut National de la Recherche Agronomique (INRA) Redon Experimental Farm, Clermont-Ferrand, France in 1996 and male sika deer moans were recorded at a private farm in Waterford, Ireland in 2007. These recordings were sampled from individuals with no known history of previous hybridization (personal communication: Rory Harrington, Marcel Verdier). See Wyman et al. (2014) for details on recording methodologies for red deer and sika deer calls. Male red × sika hybrid wails were recorded at two locations in 2007: one known F1 hybrid was recorded at the Waterford, Ireland farm while the remaining three hybrid exemplars were recorded from free-ranging males at Wicklow National Park, Ireland, a location known to contain a large population of red × sika hybrids (McDevitt et al. 2009; Smith et al. 2014). Because it was not possible to establish the initial direction of hybridization for all hybrids recorded, the term ‘red × sika hybrid’ is used here as a general term for individuals containing genetic material from both red deer and sika deer. Recordings of red × sika hybrid wails were made at distances of 8–30 m using a Sanken CS-1 directional condenser microphone (flat frequency response = 50–20,000 Hz ± 63 dB) and a Fostex FR-2 digital field recorder (amplitude resolution = 16 bits, sampling rate = 44.1 kHz). Calls from all exemplars were re-sampled to 44.1 kHz, if necessary, and normalized to 98 % of maximum intensity using Cool Edit Pro 2.0 (Syntrillium).

**Fig. 2:**
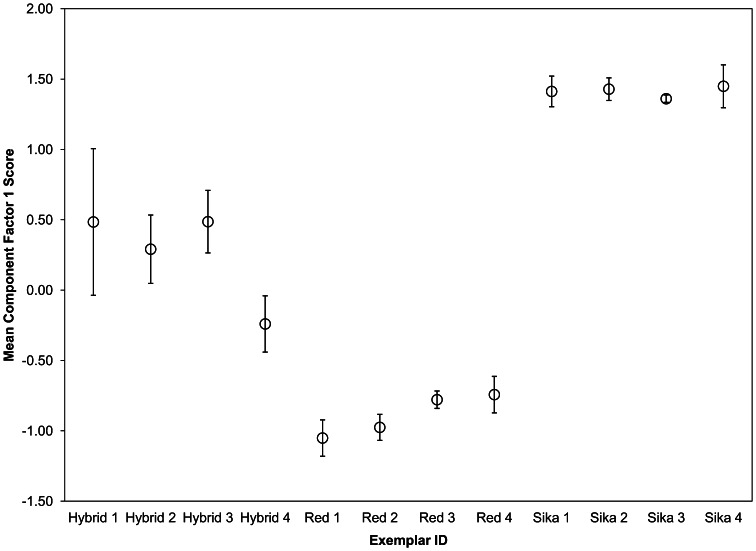
First component factor scores of acoustic parameters, averaged within exemplars. *Error bars* represent 95 % confidence intervals

General acoustic parameters were measured in all calls and compared across playback types. Call duration (s), F0 (Hz) (i.e., F0mean, F0min, F0max), and mean local variability of F0 (Hz/s) (VarF0, measured as the ‘mean absolute slope’ calculated by dividing the absolute difference in pitch values between consecutive measures in the pitch curve by the value of the time step, then averaging these values across the call, e.g., a 5 Hz difference between adjacent points with a time step of 0.01 s produces a local slope of 500 Hz/s) were measured using Praat v. 5.1.13 (Boersma and Weenink 2009) using the ‘Periodicity: To pitch’ command and the ‘Pitch Info’ query. The time step was set to 0.01 s and the pitch floor and ceiling values were set to 100–2700 Hz for sika deer, 30–180 Hz for red deer, and 100–1000 Hz for hybrid deer. Pitch floor and ceiling values were selected independently for each playback type in order to help Praat accurately track F0. The mean acoustic parameters measured from the four males selected as hybrid exemplars were strongly intermediate to the mean values for the red and sika deer exemplars (Table 1), similar to results described in Long et al. (1998). A principal component analysis (PCA) was conducted on the five measured acoustic parameters of all exemplar calls (N = 90) in order to compare the underlying structure of vocal characteristics both within and between exemplar groups. The PCA was run without rotation on log transformed parameters. Criteria for factor extraction was based on the scree plot inflection point and the amount of cumulative variance explained (>90 %). The first component (eigenvalue = 4.436) explained 88.728 % of the total variance, while the second component (eigenvalue = 0.296) added 5.914 % of explained variance, summing to 94.642 % cumulative variance. After extraction of these first two components, the first component showed strong loadings for all five acoustic parameters, with MinF0 being the weakest among them, while the second component only loaded strongly for MinF0 (Table 2). Figure 2 illustrates that the average first component factor scores of three hybrid exemplars were highly intermediate to each parent species, while one hybrid exemplar was slightly more similar to red deer scores than sika deer scores. The measured parameters were selected as broad descriptors of these deer calls and we acknowledge that there may be other unmeasured parameters present that are not fully intermediate to the parent species. However, based on this assessment and Long et al. (1998), we postulated that these calls were generally intermediate to the parent species and that the individual callers were likely to be hybrids.

**Table 1:** Acoustic profiles of male mating calls used in playback experiments (mean ± SD; range per playback stimuli type)

Acoustic parameter	Playback stimuli		
	Red deer	Sika deer	Red × sika hybrid
Call duration (s)	1.8 ± 0.4; 1.3–2.7	4.8 ± 1.0; 3.0–7.1	2.6 ± 0.7; 1.1–4.1
Mean F0 (Hz)	116.7 ± 19.9; 76.9–160.3	1045.5 ± 147.6; 753.9–1335.8	351.5 ± 127.6; 128.1–633.2
Min F0 (Hz)	72.3 ± 20.3; 40.1–122.1	244.1 ± 34.0; 183.0–357.5	197.5 ± 72.3; 120.3–386.7
Max F0 (Hz)	142.4 ± 23.0; 84.4–190.4	2094.6 ± 297.3; 1477.0–2621.7	476.2 ± 223.0; 133.6–966.8
VarF0 (Hz/s)	81.7 ± 33.8; 30.1–187.3	739.5 ± 180.2; 527.3–1172.0	240.0 ± 136.9; 76.6–592.5

**Table 2:** PCA loadings of acoustic parameters after the extraction of two components

Parameter	Component	
	1	2
LogMeanF0	0.991	0.042
LogMaxF0	0.991	−0.051
LogVarF0	0.934	−0.174
LogDuration	0.899	−0.251
LogMinF0	0.889	0.445

Male sexual calls were arranged into six bouts of similar duration, with the number of calls per bout based on the naturally observed calling behavior for each species. Red deer roar playbacks consisted of 16 calls per individual, grouped into bouts of one to four calls per bout (constructed from eight to 11 original calls, with 0.5 s between calls, and no calls used more than twice or repeated within bouts). Sika deer moan playbacks were composed of six unique single call bouts per male. Red × sika hybrid wail playbacks were comprised of 10–12 calls per male, organized into bouts of one to three calls per bout (constructed from five to six original calls with the same rules as red deer bout construction). The calling bouts and the total duration of calls per playback were constructed with similar durations between all playbacks in an effort to expose focal females to similar total durations of acoustic stimuli. The mean bout duration of red, sika, and red × sika hybrid exemplars was, respectively, 4.78, 4.84, and 4.71 s while the total call duration per playback was 28.68, 29.06, and 28.26 s.

In each playback experiment, two types of exemplars were broadcast from two speakers in paired sequences of consecutive calling bouts (e.g., red deer roars from left speaker vs. sika deer moans from right speaker). Six bouts from each exemplar were organized into matched pairs, alternating which exemplar calls first between every pair, with 2 s between the bouts within a pair and 20 s between bout pairs (see Charlton et al. 2007; Wyman et al. 2011). Randomization of the individuals used in each playback trial, the initial ‘leader’ of each playback trial, and the speaker location of each exemplar was achieved using a Latin square design. Overall, the total duration of each playback trial was approximately 3 min.

### Playback Protocol

For each species, two Anchor Liberty 6000HIC amplified speakers were hidden behind camouflage netting in the corners of a partially wooded rectangular enclosure (1040 m^2^ for red deer and 675 m^2^ for sika deer). Proximity zones were demarcated in front of the speakers and coaxial cables connected each speaker to a Toshiba NB205 netbook computer used to play the prepared playback sequences. All the females used in these experiments had previously been given free access to the playback enclosures as a feeding location. Speaker placement within the enclosures was dictated by the existing geometry and layout of these spaces, but efforts were made to make speaker and zone positions proportional between the experiments, relative to species size and enclosure space. In the red deer enclosure, speakers were positioned 6.5 m from the far wall of the arena, suspended in trees 1.5 m above the ground and 24.5 m apart. In the sika deer enclosure, the speakers were positioned against the far wall of the arena, 1 m above the ground and 14 m apart. Proximity zone boundaries were outlined with naturally occurring rocks and sticks at 14 m in front of the speakers and 12.15 m between the speakers for red deer and 8 m in front of the speakers and 7 m between the speakers for sika deer.

Only one individual was used during each playback trial. After the hind was introduced to the enclosure, the trials began when the individual was calm and near the center of the enclosure near a feeding station, equidistant from the two speakers. Calls were broadcast at amplitudes of 100 dBC SPL at 1 m to red deer and 95 dBC SPL at 1 m to sika deer, as measured by a CEM DT-805 sound level meter with C-weighting. Female behavior was video recorded using a Sony Handycam Mini DV HC52 camcorder from a hidden, elevated position.

### Behavior Coding and Statistical Analysis

Preference and attention behaviors were monitored in focal hinds in response to playback stimuli. Preference was measured as the number of instances of entering the proximity zone and the total time spent within the proximity zone in front of the speakers. Attention was measured as the number and total duration of looks directed towards the speakers. Behaviors were video recorded from the start of each playback sequence until two min post-playback and were coded using digital video analysis software Gamebreaker 7.0.121 (Sports-Tec, Sydney, Australia) at 25 fps. Hinds were defined as entering or leaving a proximity zone when their first leg crossed the zone demarcation line. Looks were operationally defined as starting when a stationary hind (or hind that stopped within two steps of look initialization) began to turn their head directly towards a speaker and ending when they began to turn their head away. Look data was not available for two trials of the red deer hind experiment and so statistical tests on this variable were only run on 16 of 18 trials. Behavioral coding was carried out by MTW and used a methodology identical to the coding process described in Wyman et al. (2011), a method which produced highly reliable results in a double coding exercise (98.3 % agreement between two coders for trials with non-zero values). The two-speaker playback experiment methodology utilized here was based on previous studies examining intraspecific mate choice decisions in female red deer (Charlton et al. 2007; Reby et al. 2010). Entrances and time spent within proximity zones in front of the speakers are clear indicators of the hind’s choice to closely approach different perceived callers and therefore are suitable operational measures of preference behaviors while in estrus.

Box plots were used to visualize the data distribution in behavioral responses, with whisker limits set at 1.5*IQR (interquartile range) of the lower and upper quartile. As the data could not be normalized for all variables, non-parametric two-tailed Wilcoxon matched-pair signed-rank tests were used to compare overall behavioral responses between the paired playbacks in each experiment. Kruskal–Wallis tests were used to check for significant differences in behavioral responses to the four individual exemplars within playback types (e.g., different responses to the four individual hybrid males). Spearman’s rank correlation test was used to determine if there was a relationship between hind preferences for stimuli type and the relative difference between the acoustic parameters of the paired playbacks. Specifically, these tests compared the difference between preference responses to the conspecific versus hybrid calls in each trial (e.g., duration in conspecific zone minus duration in hybrid zone for each trial) to the level of dissimilarity between the paired playback calls, calculated as the absolute difference between the average first component PCA factor scores for the paired exemplars from each trial. A significant positive correlation would indicate that when the difference between the paired playback calls is high, females are more likely to spend more time or number of instances in the conspecific speaker zone. Spearman’s rank correlations were also used to test for significant relationships between hind age and behavioral responses. Statistical tests were performed using SPSS (SPSS for Windows, Rel. 18.0.0. 2009. Chicago: SPSS Inc.) with 0.05 levels of significance.

## Results

### Playbacks to Red Deer Hinds

**Fig. 3:**
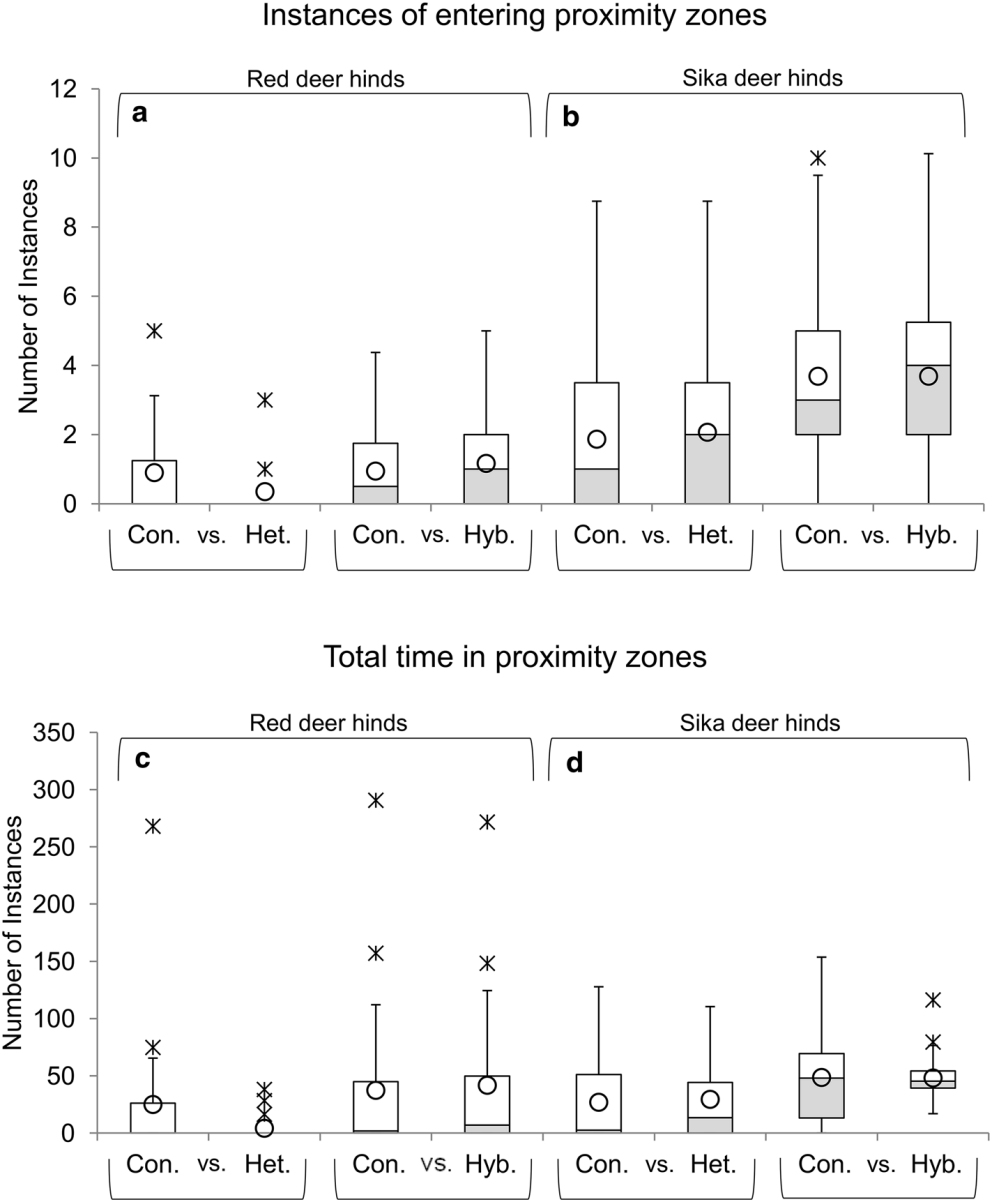
Preference behaviors exhibited by hinds in response to conspecific versus heterospecific or conspecific versus hybrid male mating calls. *Box plots* represent the preference behaviors of female red deer and sika deer: Instances (**a**) and total time (**c**) spent in proximity zones by red deer and instances (**b**) and total time (**d**) spent in proximity zones by sika deer. Data from conspecific versus heterospecific playback experiments are sourced from Wyman et al. 2011 (red deer hinds) and Wyman et al. 2014 (sika deer hinds) for comparison with the conspecific versus hybrid experiments. *Upper* and *lower* whisker limits are set to 1.5*IQR (interquartile range) above and below the third and first quartile, respectively. *Circles* represent mean preference behavior values and *stars* represent outliers present outside of the whisker limits

Thirteen of 18 (72.22 %) red deer hinds entered at least one proximity zone during this experiment. While some hinds preferred conspecific zones, others preferred hybrid zones (Table 3), resulting in similar preference variability in response to these two call types (conspecific/hybrid: IQR of entering zones = 1.75/2.0, IQR of duration in zones = 44.84/49.78 s; Table 4; Fig. 3a, c). Furthermore, there were no significant differences in the number of instances that the hinds entered the two proximity zones (z_18_ = −0.47, *P* = 0.640) or in the total time spent within these zones (z_18_ = −0.66, *P* = 0.507) (Table 4). Although the scale of behavioral measures are similar between the conspecific versus hybrid trials shown here and the conspecific versus heterospecific trails from Wyman et al. 2011 (see Fig. 3), female red deer tend to exhibit slightly more preference behaviors to both stimuli types during the conspecific versus hybrid trials. There were no significant differences in the number of looks (z_16_ = −0.06, *P* = 0.95) or total duration of looks (z_16_ = −0.21, *P* = 0.836) directed towards either call type (Table 4).

**Table 3:** Proximity zone preference by individual red deer and sika deer hinds that entered at least one proximity zone during trials

Behavioral measure	Proximity zone preference	Red deer hinds	Sika deer hinds
Instances of entering zone	Conspecific	6 of 13 (46.16 %)	6 of 16 (37.50 %)
	Hybrid	7 of 13 (53.85 %)	6 of 16 (37.50 %)
	Equal	–	4 of 16 (25.00 %)
Total time spent in zone	Conspecific	4 of 13 (30.77 %)	8 of 16 (50.00 %)
	Hybrid	9 of 13 (69.23 %)	8 of 16 (50.00 %)
	Equal	–	–

The proximity zone preference of individual hinds was defined as the zone with the higher number of zone entrances or total time spent within zones during each trial. Data represent the number and percentage of hinds per experiment type that exhibited more preference behaviors (instances and total time) within particular zones, or an equal number of instances entering within each zone type

**Table 4:** Behavioral responses by female red deer and sika deer to conspecific versus hybrid male mating calls

Behavioral measure	Focal species	Conspecific stimuli	Hybrid stimuli	Wilcoxon test
Instances of entering zone	Red deer	0.50, 0–4.00 (1.75)	1.00, 0–4.00 (2.00)	Z = −0.47, *P* = 0.64
	Sika deer	3.00, 0–10.00 (3.00)	4.00, 0–7.00 (3.25)	Z = −0.16, *P* = 0.872
Total time in zone (s)	Red deer	1.74, 0–290.69 (44.84)	6.94, 0–271.69 (49.78)	Z = −0.66, *P* = 0.507
	Sika deer	48.04, 0–151.08 (56.19)	45.26, 0–116.21 (14.90)	Z = −0.05, *P* = 0.959
Instances of looking at speaker	Red deer	5.00, 0–14.00 (4.75)	5.00, 0–10.00 (5.25)	Z = −0.06, *P* = 0.95
	Sika deer	4.00, 1.00–12.00 (5.00)	4.50, 2.00–12.00 (7.25)	Z = −0.43, *P* = 0.668
Total time looking at speaker (s)	Red deer	12.39, 0–46.31 (16.73)	13.35, 0–24.71 (13.58)	Z = −0.21, *P* = 0.836
	Sika deer	8.20, 1.40–35.60 (12.38)	9.75, 1.10–24.30 (7.93)	Z = −0.62, *P* = 0.535

Behavioral responses were summarized using median, range, and interquartile range values [Mdn, range (IQR^a^)]. Wilcoxon signed rank tests (Z score, *P* value) were used to test for significant differences in female responses to the conspecific and hybrid stimuli

^a^IQR: Interquartile range of behavioral responses (range of the middle 50 % of behavioral scores) calculated as the difference between the third quartile and first quartile

No significant differences were found between the mean rank of female red deer preference responses (i.e., instances/duration in zones) to individual exemplars within playback types (Table 5), meaning that the red deer hinds did not show more preference behaviors towards a particular exemplar male compared to the other exemplar males of that type (i.e., conspecific or hybrid type). However, there was a significant difference in the mean rank of the number of looks given towards the hybrid speaker in response to the hybrid exemplars (H(3) = 9.950, *P* = 0.019), although none of the pair-wise comparisons between hybrid exemplars were significant after Bonferroni corrections.

**Table 5:** Comparison of female responses to exemplars within playback type

Behavioral measure	Speaker playback stimuli	Focal species	Exemplar playback type	
			Conspecific	Hybrid
Instances of entering zone	Conspecific	Red deer	H = 4.563, *P* = 0.207	H = 0.449, *P* = 0.930
		Sika deer	H = 0.454, *P* = 0.929	H = 0.454, *P* = 0.929
	Hybrid	Red deer	H = 4.390, *P* = 0.222	H = 1.685, *P* = 0.640
		Sika deer	H = 0.767, *P* = 0.857	H = 0.767, *P* = 0.857
Total time in zone	Conspecific	Red deer	H = 3.891, *P* = 0.273	H = 0.535, *P* = 0.911
		Sika deer	H = 0.370, *P* = 0.946	H = 0.370, *P* = 0.946
	Hybrid	Red deer	H = 2.232, *P* = 0.526	H = 1.157, *P* = 0.763
		Sika deer	H = 0.635, *P* = 0.888	H = 0.635, *P* = 0.888
Instances of looking at speaker	Conspecific	Red deer	H = 1.372, *P* = 0.712	H = 3.182, *P* = 0.364
		Sika deer	H = 1.468, *P* = 0.690	H = 1.468, *P* = 0.690
	Hybrid	Red deer	H = 3.383, *P* = 0.336	H = 9.950, *P* = 0.019
		Sika deer	H = 3.105, *P* = 0.376	H = 3.105, *P* = 0.376
Total time looking at speaker	Conspecific	Red deer	H = 0.325, *P* = 0.955	H = 1.089, *P* = 0.780
		Sika deer	H = 1.204, *P* = 0.752	H = 1.204, *P* = 0.752
	Hybrid	Red deer	H = 2.960, *P* = 0.398	H = 4.616, *P* = 0.202
		Sika deer	H = 4.867, *P* = 0.182	H = 4.867, *P* = 0.182

Comparisons of female behavioral responses to the four individual exemplars within playback types were tested using Kruskal–Wallis tests (Chi square value, *P* value) with exemplar ID as the grouping variable (*df* = 3)

No significant correlations were found between the call dissimilarity of paired playbacks and the difference between the number of times hinds entered the conspecific versus the hybrid zone (r_s 18_ = −0.079, *P* = 0.755) or the total duration of time spent in the conspecific versus the hybrid zone (r_s 18_ = 0.038, *P* = 0.882). Furthermore, red deer hind age did not correlate with behavioral responses (Table 6).

**Table 6:** Correlations between female age and behavioral responses to playback stimuli

Behavioral measure	Focal species	Conspecific stimuli	Hybrid stimuli
Instances of entering zone	Red deer	r_s 16_ = −0.001, *P* = 0.996	r_s 16_ = −0.001, *P* = 0.998
	Sika deer	r_s 14_ = 0.267, *P* = 0.317	r_s 14_ = 0.436, *P* = 0.091
Total time in zone	Red deer	r_s 16_ = −0.067, *P* = 0.790	r_s 16_ = 0.190, *P* = 0.451
	Sika deer	r_s 14_ = 0.022, *P* = 0.935	r_s 14_ = 0.131, *P* = 0.629
Instances of looking at speaker	Red deer	r_s 14_ = −0.153, *P* = 0.571	r_s 14_ = −0.197, *P* = 0.464
	Sika deer	r_s 14_ = 0.156, *P* = 0.564	r_s 14_ = 0.046, *P* = 0.867
Total time looking at speaker	Red deer	r_s 14_ = −0.021, *P* = 0.939	r_s 14_ = −0.194, *P* = 0.473
	Sika deer	r_s 14_ = 0.149, *P* = 0.581	r_s 14_ = 0.131, *P* = 0.628

Correlations were measured using the Spearman rank test (r_s df_, *P* value)

### Playbacks to Sika Deer Hinds

All 16 sika deer hinds entered a proximity zone at least once during the course of their trial with equal numbers of hinds displaying more preference behaviors within the conspecific zone and within the hybrid zone (Table 3). Although the variability in the number of instances hinds entered the zones was similar (conspecific/hybrid IQR = 3.0/3.25), sika hinds displayed more variability in the total duration spent in conspecific zones than hybrid zones (conspecific/hybrid IQR = 56.19/14.90 s) (Table 4; Fig. 3b, d). However, there were no overall significant differences in the number of times hinds entered the two proximity zones (z_16_ = −0.16, *P* = 0.872) or in the total time spent within the two proximity zones (z_16_ = −0.05, *P* = 0.959) (Table 4). As seen in red deer, the response of female sika deer to conspecific versus hybrid stimuli is on a similar scale to their responses to conspecific versus heterospecific stimuli (Wyman et al. 2014), although with a slight trend for more behaviors directed at both stimuli for the conspecific versus hybrid trials (Fig. 3). Furthermore, there were no significant differences in the number of looks (z_16_ = −0.43, *P* = 0.668) or total duration of looks (z_16_ = −0.62, *P* = 0.535) towards either call type (Table 4).

There were no significant differences in the mean rank of female sika deer responses to individual exemplars within playback types (Table 5). Similar to red deer hinds, there were no significant correlations between call dissimilarity of paired playbacks and the difference between the number of times sika deer hinds entered the conspecific versus the hybrid zone (r_s 16_ = −0.005, *P* = 0.986) or the difference between the total amount of time spent in each zone (r_s 16_ = 0.002, *P* = 0.996). Additionally, no significant correlations were present between sika deer hind age and behavioral responses (Table 6).

## Discussion

In this study, we investigated the role of male mating calls in the sexual isolation between polygynous deer species and their hybrids within the early stages of secondary contact between previously allopatric populations. We predicted that the asymmetry in preference behaviors previously documented between estrous red deer and sika deer hinds in response to male mating calls from unfamiliar conspecifics versus novel reproductively compatible heterospecifics (Wyman et al. 2011, 2014) would persist when species were presented with mating calls from unfamiliar conspecific males versus novel red × sika hybrid males. However, the results indicate that no asymmetry remained; neither focal species displayed significantly different preference behaviors towards speakers broadcasting conspecific or hybrid male mating calls (Table 4; Fig. 3). While red deer hinds appear to be strongly deterred by sika deer calls, this did not persist with hybrid deer calls as they showed similar variability in preference behaviors directed at conspecifics versus hybrids. In comparison, sika deer hinds were not strongly deterred by either type of non-conspecific call. We acknowledge that larger sample sizes may reduce the chance of type II errors, however, similar sample sizes produced significant results in related studies of female preference behaviors (Charlton et al. 2007; Reby et al. 2010; Wyman et al. 2011). Furthermore, preferences for non-conspecific calls displayed by even a small proportion of estrous hinds is important as this could have large evolutionary implications in free-living populations. Additionally, female red deer and sika deer did not show differences in preference behaviors between individuals within exemplar types (Table 5) or differences in preference behaviors in relation to dissimilarity between paired playback stimuli. Overall, these results indicate that the variance within exemplar group and the variance in dissimilarity between paired calls is small relative to the dissimilarity between exemplar groups. Similar to previous playback experiments (Wyman et al. 2011, 2014), both focal species displayed no significant difference in attention behaviors (i.e., number and duration of looks) directed towards the speakers (Table 4), demonstrating that mating calls from unfamiliar conspecific males do not elicit significantly more attention from females than novel male mating calls from hybrids. Furthermore, no significant relationships were found between hind age and behavioral responses (Table 6).

Mammalian sexual calls have the potential to present a strong barrier to hybridization and introgression as they are largely non-learned, anatomically constrained, and stereotypical within a species. These barriers should be especially strong in species that actively use sexually selected vocalizations during mate choice decisions as receivers carefully attend to signal parameters. Despite these characteristics, some female red deer and sika deer showed preference behaviors for an unexpectedly wide range of signal parameters within male mating calls. Given the large differences between the mating calls of these species, the intermediate hybrid calls are still quite acoustically different from either parent species. However, these hybrid calls do not appear to present a solid barrier to gene flow in either species as some estrous females of both species closely approached the hybrid speaker over the conspecific speaker (Table 3), a behaviour which would increase the chances of introgression in the free-living animals. In the wild, the movement of females between harems or territories is not uncommon in red deer (Clutton-Brock et al. 1982; Carranza et al. 1996) or sika deer (Endo and Doi 2002; Minami et al. 2009). In sika deer, males typically defend territories that females move through. Observations of female ‘escape’ behaviors prompted Endo and Doi (2002) to suggest that sika deer hinds move away from subordinate males to gain a mate of higher quality. Stopher et al. (2011) found that estrous red deer hinds were more likely to change harems, and travel longer distances to do so, than non-estrous hinds. Furthermore, approximately 45 % of harem changes during estrus resulted in the male of the new harem siring the hind’s offspring. Overall, females were more likely to move to harems with a younger male or larger size than their previous harem, but these preferences did not persist when only examining estrous hinds (Stopher et al. 2011). However, it was acknowledged that other untested male phenotypes, such as particular roar parameters, may impact female movements and harem preference. In the context of the present study, it is therefore very feasible that free-living female red deer or sika deer may move towards hybrid vocalizations, and thus, run the risk of an introgressive mating, if some element of the call induces approach behaviors.

Overall, the results presented here show that intermediate hybrid mating signals can inhibit species discrimination abilities in the early stages of sympatry between species, even in a species that actively discriminates against heterospecific mating signals. Reduced discrimination against hybrid signals compared to signals from reproductively compatible heterospecifics is documented in other taxa, such as anurans (Gerhardt 1974; Höbel and Gerhardt 2003), fish (van der Sluijs et al. 2008), and birds (Derégnaucourt and Guyomarc’h 2003). Similar results were reported by Rosenfield and Kodric-Brown (2003) regarding mate choice within *Cyprinodon variegatus* (sheepshead minnows) and *C. pecosensis* (Pecos pupfish); despite asymmetries in females preference responses to visual presentations of purebred males (*C. pecosensis* preferred male *C. variegatus* over conspecifics while *C. variegatus* showed no preference), neither species discriminated against F1 hybrid males.

In the context of our experiments, why would estrous females approach novel hybrid calls and risk potentially reduced reproductive success when conspecific calls are being concurrently presented? These unexpected mate choice decisions may be rooted in several alternative explanations.

### Signal Attraction

If non-conspecific signals are similar enough to conspecific signals to pass initial species discrimination tests (i.e., comparison of received signals against a phenotypic template of conspecific signals), attraction towards these signals may occur if they contain novel (Arak and Enquist 1993; Elias et al. 2006) or exaggerated (Ryan and Keddy-Hector 1992; Searcy 1992; Randler 2002) elements that neurologically trigger approach behaviors. For example, current intraspecific selection pressures that result in directional preferences for particular signal parameters may result in attraction towards non-conspecific signals that contain exaggerations of these parameters (Rosenfield and Kodric-Brown 2003; Meyer et al. 2006), especially within the early stages of secondary contact between populations. While red deer hinds displayed a strong aversion to heterospecific calls (Wyman et al. 2011), slightly more than half of the hinds tested in this study entered or spent more total time in the hybrid zone over the conspecific zone (Table 3). This observed interest in the intermediate hybrid calls demonstrated by some red deer hinds may be the result of existing intraspecific selection pressures as previous experiments demonstrated that estrous red deer hinds prefer conspecific male mating calls with higher F0 over lower F0 (Reby et al. 2010). Therefore, some red deer hinds may be attracted to the exaggerated higher-pitch elements present within the hybrid vocalizations. In comparison, the much higher pitched sika deer calls may exceed this upper preference window in red deer hinds, prompting strong species discrimination against these calls. The potential conflict between species and mate-quality recognition is well established (Ryan and Rand 1993a; Pfennig 1998). In this case, the intermediate hybrid signals may generate this type of conflict by both reducing species recognition and activating preference behaviors in estrous female red deer.

Sika deer hinds were not strongly deterred by either type of non-conspecific call. An equal number of hinds entered or spent more time in the hybrid zone as hinds who entered or spent more time in the conspecific zone. Female Japanese quail, *Coturnix coturnix japonica*, exhibit a similar lack of discrimination in response to male calls from conspecifics, closely related species (*C. c. coturnix*), and hybrids (Derégnaucourt and Guyomarc’h 2003). Acceptance of a wide range of mating signal parameters from potential mates is also documented in other hybridizing species (e.g., Gerhardt 1974; Littlejohn and Watson 1976; Smadja and Ganem 2002; Gee 2005). In sika deer hinds, the demonstration of preference responses to a broad spectrum of signal parameters may be linked to the partially coupled evolution of female preferences and the wide repertoire of acoustic signals produced by sika deer stags (Minami and Kawamichi 1992).

The lack of discrimination against hybrids observed in some sika deer hinds may also potentially stem from pre-existing sensory biases that could result in attraction behaviors to ancestral signal elements from closely related species (Ryan and Rand 1993b; Hill 1994; Endler and Basolo 1998). The higher pitched calls of sika deer appear to be a derived characteristic within *Cervidae* (Cap et al. 2008), with the most recent common ancestor between red deer and sika deer likely possessing a lower pitched call. As such, although male sika deer calls have evolved a higher pitch, female sika deer may still retain some pre-existing sensory biases that are triggered by the lower pitched elements present within red deer roars and intermediate hybrid wails. This type of partially decoupled evolution between male signals and female preference was offered as a possible explanation by Smadja and Ganem (2002) for asymmetries in subspecies discrimination of male olfactory signals by female *Mus mus domesticus* and *M. m. musculus*.

Although a relatively large proportion of estrous red deer and sika deer hinds did not discriminate strongly between conspecifics and hybrids based the particular signal types presented, these deer may employ additional signals (i.e., other acoustic, chemical, or visual signals) or a combination of signals during species and mate choice assessments (Pfennig 1998; Candolin 2003; Hankison and Morris 2003). As such, females may show stronger discrimination against non-conspecifics when presented with additional signal types.

### Species Familiarity

The history of contact between the species or populations will also impact mate preference decisions involving non-conspecific mating signals (Pfennig 1998). Closely related sympatric species or populations tend to have stronger species discrimination abilities than closely related allopatric species or populations (Coyne and Orr 1989; Gerhardt 1994; Höbel and Gerhardt 2003). As allopatric species become more sympatric, species discrimination abilities are likely to increase due to factors such as reproductive character displacement and reinforced species recognition, if hybridization is maladaptive (Noor 1999; Höbel and Gerhardt 2003; Coyne and Orr 2004; Pfennig and Pfennig 2005). However, mate selection preferences may evolve to favor non-conspecific mates under certain circumstances if hybridization results in fitness benefits (Veen et al. 2001; Pfennig and Simovich 2002; Mallet 2007).

Our experiments examined the early stages of species interactions between individuals from naturally allopatric species who were naïve to hybrid vocalizations. Based on molecular analyses, the most recent common ancestor of *C. e. scoticus* and *C. n. nippon* began diverging approximately 3.5 (Pitra et al. 2004) to 7 million years ago (Ludt et al. 2004) in Central Asia, with the ancestors of *C. e. scoticus* and *C. n. nippon* moving westward and eastward, respectively (Ludt et al. 2004). In locations where the distributions of *C. e. scoticus* and introduced *C. n. nippon* have overlapped in Kintyre Peninsula, Scotland, hybridization is rare while introgression can be widespread within certain locations (Senn and Pemberton 2009; Senn et al. 2010a). Nuclear and mitochondrial DNA evidence suggest that female red deer were strongly involved in initial hybridization and backcrossing with male sika deer and hybrids, although other types of crosses did occur such as interbreeding among hybrids. Similar results were found in most sympatric populations of *C. elaphus* and *C. n. nippon* in Ireland (McDevitt et al. 2009; Smith et al. 2014) and Eastern Europe (Biedrzycka et al. 2012). Although the direction of these crosses may be influenced by several factors, such as differences in morphology (e.g., red deer are approximately twice the size of sika deer) and local species densities (e.g., dispersal of male sika deer into red deer-dominated locations, Senn et al. (2010a), or the flood of sika deer herds into sparsely populated red deer ranges, Smith et al. 2014), sexual behavior involving mate choice decisions are also likely to play a strong role in interbreeding. The relatively higher levels of introgression may, in part, be a consequence of the weak discrimination against novel hybrid male mating signals demonstrated by both species in this study.

As local interactions between red deer, sika deer, and their hybrids increase over time, species discrimination abilities and the direction and likelihood of hybridization and introgression will be strongly influenced by both endogenous (genetic-based) and exogenous (environmental-based) selection pressures on adaptive mate choice and hybrid fitness (Noor 1999; Wirtz 1999; Burke and Arnold 2001; Veen et al. 2001; Gee 2003; Rieseberg et al. 2003; Seehausen 2004; Arnold et al. 2008). Increased discrimination against hybrid signals may evolve through reinforced species discrimination and character displacement if interbreeding brings fitness costs. Phenotypic analysis in relation to introgression levels in the Scottish populations revealed no significant difference in kidney fat weight or pregnancy rates between hybrids and the pure species they were most similar to genetically (Senn et al. 2010b). However, hybridization did significantly affect various mass and size measures (e.g., increased jaw length in sika-like hybrid females, increased carcass weight in sika-like hybrid males). These physical effects may potentially carry both ecological and reproductive consequences (e.g., altering foraging behaviors, ability to defend a harem, vocal production anatomy, etc.) that influence the evolution of mating preferences in these species (Turelli et al. 2001; Pfennig 2009). Ultimately, more research is needed to investigate the potential positive or negative impacts of interbreeding and the evolution of preference behaviors in these species within hybrid zones.

## Conclusion

This study illustrates the impact that hybrid vocalizations may have on further introgression between previously allopatric populations during recent secondary contact. Intermediate sexual signals of hybrids can negate previous asymmetries in species discrimination abilities between species. Furthermore, the high variability in both sika deer and red deer hind preference responses to novel hybrid calls indicate that hybrid vocalizations may help facilitate introgression between hybrids and both parent species. Additional studies on the source of inter-individual variation in species discrimination demonstrated during this study would be highly beneficial.

Examining the interplay of species discrimination, hybrid signals, and mate choice is important for a greater understanding of key evolutionary mechanisms such as introgression and assortative mating and their implications for future biodiversity. Anthropogenic influences, such as increased habitat alteration, introductions, and climate change, will continue to shift species distributions and increase contact between previously allopatric species and populations, resulting in higher rates of hybridization and introgression (Hoffmann and Sgrò 2011). This underlines the crucial need to understand more about the mechanisms that may promote or impede these important evolutionary processes, especially in mammals, an understudied taxon in this field.

## Electronic supplementary material

Below is the link to the electronic supplementary material.

Supplementary material 1 (MPG 1650 kb)
